# O.4.6-8 Dutch school policies for less sitting and more physical activity in primary schools

**DOI:** 10.1093/eurpub/ckad133.227

**Published:** 2023-09-11

**Authors:** Joske Nauta, Mandy Schweitzer, Ilse Kat, Kirsten Moll, Kick Koenders, Mirka Janssen

**Affiliations:** Amsterdam University of Applied Sciences, The Netherlands; j.nauta@hva.nl; Amsterdam University of Applied Sciences, The Netherlands; j.nauta@hva.nl; Amsterdam University of Applied Sciences, The Netherlands; j.nauta@hva.nl; Amsterdam University of Applied Sciences, The Netherlands; j.nauta@hva.nl; Amsterdam University of Applied Sciences, The Netherlands; j.nauta@hva.nl; Amsterdam University of Applied Sciences, The Netherlands; j.nauta@hva.nl

## Abstract

**Purpose:**

A more dynamic school day that includes less sitting and more physical activity throughout the day may support health in primary school children. In this mixed methodologies study, we assessed which physical activity possibilities are incorporated in the school policies of Dutch primary schools and if this translates into more physical activity in pupils.

**Methods:**

Seven primary schools were included in the study. The head teachers completed a questionnaire on the incorporation of physical activity in the school policy. Physical activity policy was assessed for physical education, physical activity during breaks, scheduling of physical activity during lessons, after school physical activity and active transport to school. In each school, actual physical activity was objectively measured using accelerometers in pupils in two classes and planning and logging of PA by teachers.

**Results:**

We will assess the impact of the school policy on physical activity in pupils using logistic regression. We will assess the impact of the several school policies on total physical activity in pupils, and on the three (light, moderate and high) modalities of physical activity.

**Conclusion:**

The results of this study will give us more insight in the current role of physical activity in Dutch primary schools. The potential impact of the school policy on physical activity levels in pupils can be used as a starting point for a more integral dynamic school day approach.

**Support/Funding Source:**

This study was partially funded by “Stichting Westelijke Tuinsteden’.

